# Supplementary material for: Epigenetic analysis of Paget’s disease of bone identifies differentially methylated loci that predict disease status
Source: eLife. 2021 Apr 30;10:e65715. doi: 10.7554/eLife.65715 (PMC8184208; doi:10.7554/eLife.65715)
Supplement: Transparent reporting form [file elife-65715-transrepform.docx]

***eLife’s* transparent reporting form**

We encourage authors to provide detailed information *within their submission* to facilitate the interpretation and replication of experiments. Authors can upload supporting documentation to indicate the use of appropriate reporting guidelines for health-related research (see [EQUATOR Network](http://www.equator-network.org/%20)), life science research (see the [BioSharing Information Resource](https://biosharing.org/)), or the [ARRIVE guidelines](http://www.plosbiology.org/article/info:doi/10.1371/journal.pbio.1000412) for reporting work involving animal research. Where applicable, authors should refer to any relevant reporting standards documents in this form.

If you have any questions, please consult our Journal Policies and/or contact us: [editorial@elifesciences.org](mailto:editorial@elifesciences.org).

**Sample-size estimation**

- You should state whether an appropriate sample size was computed when the study was being designed
- You should state the statistical method of sample size computation and any required assumptions
- If no explicit power analysis was used, you should describe how you decided what sample (replicate) size (number) to use

Please outline where this information can be found within the submission (e.g., sections or figure legends), or explain why this information doesn’t apply to your submission:

# We based our power calculation on the results from the study by Tsai and Bell entitled ‘Power and sample size estimation for epigenome-wide association scans to detect differential DNA methylation’ where it was found that a 10% effect size at genome-wide significance threshold of P = 1 × 10^−6^ requires 112 samples from cases and controls to reach 80% EWAS power.

# This information is indicated in the manuscript in the methods section at the end of the subsection ‘study subjects’ on page 10.

**Replicates**

- You should report how often each experiment was performed
- You should include a definition of biological versus technical replication
- The data obtained should be provided and sufficient information should be provided to indicate the number of independent biological and/or technical replicates
- If you encountered any outliers, you should describe how these were handled
- Criteria for exclusion/inclusion of data should be clearly stated
- High-throughput sequence data should be uploaded before submission, with a private link for reviewers provided (these are available from both GEO and ArrayExpress)

Please outline where this information can be found within the submission (e.g., sections or figure legends), or explain why this information doesn’t apply to your submission:

The section under the subheading ‘DNA methylation profiling’ in the methods section (page 10) has a detailed description of the QC preprocessing analysis of the measured samples and criteria of exclusion. The study does not feature any technical replicates. Only Biological replicates consisting of separate individuals were included in the cases and controls. The dataset was randomly split into discovery and replication.

Processed and Raw methylation data as well as phenotype data were uploaded to GEO under the accession number GSE163970. Data will be visible to public on publication of the manuscript. In the meantime, reviewers can go to <https://www.ncbi.nlm.nih.gov/geo/query/acc.cgi?acc=GSE163970> and use the token cfqvgiacvfiflmn to access the data.

**Statistical reporting**

- Statistical analysis methods should be described and justified
- Raw data should be presented in figures whenever informative to do so (typically when N per group is less than 10)
- For each experiment, you should identify the statistical tests used, exact values of N, definitions of center, methods of multiple test correction, and dispersion and precision measures (e.g., mean, median, SD, SEM, confidence intervals; and, for the major substantive results, a measure of effect size (e.g., Pearson's r, Cohen's d)
- Report exact p-values wherever possible alongside the summary statistics and 95% confidence intervals. These should be reported for all key questions and not only when the p-value is less than 0.05.

Please outline where this information can be found within the submission (e.g., sections or figure legends), or explain why this information doesn’t apply to your submission:

Requested information can be found in the ‘statistics.xlsx’ file uploaded with the manuscript.

(For large datasets, or papers with a very large number of statistical tests, you may upload a single table file with tests, Ns, etc., with reference to sections in the manuscript.)

**Group allocation**

- Indicate how samples were allocated into experimental groups (in the case of clinical studies, please specify allocation to treatment method); if randomization was used, please also state if restricted randomization was applied
- Indicate if masking was used during group allocation, data collection and/or data analysis

Please outline where this information can be found within the submission (e.g., sections or figure legends), or explain why this information doesn’t apply to your submission:

PDB patients were allocated to cases on the basis of medical examination (more details of the test performed can be found under ‘Study subjects’ in Materials and Methods.

Allocation of samples to discovery or replication sets was random.

**Additional data files (“source data”)**

- We encourage you to upload relevant additional data files, such as numerical data that are represented as a graph in a figure, or as a summary table
- Where provided, these should be in the most useful format, and they can be uploaded as “Source data” files linked to a main figure or table
- Include model definition files including the full list of parameters used
- Include code used for data analysis (e.g., R, MatLab)
- Avoid stating that data files are “available upon request”

Please indicate the figures or tables for which source data files have been provided:

All sites and regions with a differential methylation effect replicated at the FDR level < 0.05 are listed in separate tables in the supplementary material. The statistical models for site and region analyses are described in full on pages 11&12 in the Materials and Methods section. Clinical information and the demographics of the cohort as well as processed methylation data can be found at GEO under accession GSE163970.
